# Expression defect of the rare variant/Brugada mutation R1512W depends upon the SCN5A splice variant background and can be rescued by mexiletine and the common polymorphism H558R

**DOI:** 10.1080/19336950.2021.1875645

**Published:** 2021-02-04

**Authors:** Rou-Mu Hu, Evelyn J. Song, David J. Tester, Isabelle Deschenes, Michael J. Ackerman, Jonathan C. Makielski, Bi-Hua Tan

**Affiliations:** a Department of Cardiology, Heart Center & Beijing Key Laboratory of Hypertension, Beijing Chaoyang Hospital, Capital Medical University, Beijing, China; b Division of Cardiovascular Medicine, Department of Medicine, University of Wisconsin, Madison, WI, USA; c Department of Medicine, Johns Hopkins University School of Medicine, Baltimore, MD, USA; d Departments of Medicine, Pediatrics, and Molecular Pharmacology and Experimental Therapeutics, Mayo Clinic, Rochester, MN, USA; e Department of Physiology and Cell Biology, The Dorothy M. Davis Heart and Lung Research Institute, Frick Center for Heart Failure and Arrhythmia, the Ohio State University, Columbus, OH, USA

**Keywords:** SCN5A, splice variant, mexiletine, common polymorphism

## Abstract

***Background***: Mutations in SCN5A that decrease Na current underlie arrhythmia syndromes such as the Brugada syndrome (BrS). *SCN5A* in humans has two splice variants, one lacking a glutamine at position 1077 (Q1077del) and one containing Q1077. We investigated the effect of splice variant background on loss-of-function and rescue for R1512W, a mutation reported to cause BrS.***Methods and results***: We made the mutation in both variants and expressed them in HEK-293 cells for voltage-clamp study. After 24 hours of transfection, the current expression level of R1512W was reduced by ~50% in both Q1077del and Q1077 compared to the wild-type (WT) channel, respectively. The activation and inactivation midpoint were not different between WT and mutant channels in both splice variant backgrounds. However, slower time constants of recovery and enhanced intermediate inactivation were observed for R1512W/Q1077 compared with WT-Q1077, while the recovery and intermediate inactivation parameters of R1512W/Q1077del were similar to WT-Q1077del. Furthermore, both mexiletine and the common polymorphism H558R restored peak sodium current (*I*_Na_) amplitude of the mutant channel by increasing the cell surface expression of SCN5A.***Conclusion***: These findings provide further evidence that the splice variant affects the molecular phenotype with implications for the clinical phenotype, and they provide insight into the expression defect mechanisms and potential treatment in BrS.

## INTRODUCTION

The *SCN5A* gene encodes the voltage-gated cardiac Na^+^ channel (Na_v_1.5) also denoted SCN5A which is responsible for generating a large peak inward Na current (*I*_Na_) that underlies excitability and conduction in the working myocardium and specialized conduction tissue [1]. SCN5A consists of a pore-forming α-subunit composed of four homologous domains (I–IV), each containing six transmembrane segments (S1-S6). Alternative splicing of a glutamine (Q) at the beginning of exon 18 causes insertion of glutamine at position 1077 (Q1077), resulting in two splice variants, one containing 2,016 amino acids that are designated Q1077 and one lacking this glutamine that is designated Q1077del [2–4]. Messenger RNA for these two splice variants was present in every human heart examined at a ratio of approximately 2:1 with the shorter 2,015-amino acid variant Q1077del predominant [2,3]. The longer and less abundant Q1077 background has been used in most previous studies of mutations in SCN5A [5].

Mutations in SCN5A that pathologically decrease peak *I*_Na_, i.e. “loss-of-function” effect, cause phenotypes including Brugada Syndrome (BrS), progressive cardiac conduction disease, and congenital sick sinus syndrome [6,7]. Loss-of-function can occur because of altered channel gating or decreased expression of channels at the cell surface [8,9]. R1512W was reported previously as a BrS mutation [10] and a genetic cause of Chinese sudden unexplained nocturnal death syndrome (SUNDS) [11]. The R1512W variant has been also observed in 14 out of 125,636 individuals in the overall population and more specifically in 10 out of 17,265 Latinos in the genome aggregation database (gnomAD) [12]. A single base transition (C→T) at position 4534 (CGG→TGG) in exon 26 resulted in a replacement of arginine by a tryptophan at codon 1512 within the III–IV linker of the sodium channel. An important contributor to the variable genotype–phenotype relationships of genetic disorders is the genetic background such as single-nucleotide polymorphisms. H558R (c.1673, A > G, *rs1805124*) is by far the most common SCN5A polymorphism and this variant is present in all four ethnic groups, albeit at a significantly lower prevalence in Asians [2,13]. It has become clear that the H558R polymorphism can either mitigate [14,15] or aggravate [16,17] the effects of specific mutations in SCN5A. Previously, we demonstrated that the molecular phenotype of the SCN5A missense mutations G1406R and S1787N depends upon the splice variant background in which it is expressed [3,18]. We hypothesized that splice variant background might also affect other mutations, causing loss-of-function, and this expression defect can be modulated by the common polymorphism H558R and drugs.

## METHODS

### Site-directed mutagenesis and heterologous expression

The R1512W mutation was created by site-directed mutagenesis (mutagenesis kit from Stratagene) using a PCR technique. The appropriate nucleotide changes for R1512W were engineered into two common splice variant backgrounds, one lacks glutamine at position 1077 (Q1077del; GenBank accession no. AY148488) and the other includes Q1077 (Q1077; GenBank accession no. AC1377587) [2–4], as well as the most common splice variant background Q1077del with a common polymorphism H558R in the pcDNA3 vector (Invitrogen, Carlsbad, CA). The integrity of the constructs was verified by DNA sequencing to confirm the presence of the introduced mutation and to exclude the possibility of other substitutions that may occur during PCR. The cDNA of wide-type (WT), R1512W or R1512W/H558R in these two splice variant backgrounds and another plasmid containing the construct for the green fluorescent protein (GFP) were transiently co-transfected into HEK 293 cells at a ratio of 5:1 with Superfect (Qiagen, Valencia, CA) following manufacturer’s recommended protocol. After 3–5 hours of incubation, the transfection reagent-DNA mixture was replaced with 3 ml of normal culture medium with or without 500 μM mexiletine, and the cells were incubated at 37°C for 24 hours. Before the electrophysiological recording, the plates containing the cells were removed from the 37°C incubator, growth medium was aspirated off the plates with or without the drug, and the cells were treated with a 0.25% trypsin-1 mM EDTA solution (GIBCO-BRL) and transferred to a fresh tube along with 2 ml of normal culture medium and directly to the experimental chamber for electrophysiological recording.

### Standard electrophysiological measurements

Macroscopic voltage-gated *I*_Na_ was measured 24 hours after transfection with the standard whole-cell patch-clamp technique at a temperature of 22–24°C in HEK 293 cells expressed the GFP. Cells were continuously perfused with bath (extracellular) solution containing 140 mM NaCl, 4 mM KCl, 1.8 mM CaCl2, 0.75 mM MgCl2 and 5 mM HEPES (pH 7.4 set with NaOH). The pipette (intracellular) solution contained 120 mM CsF, 20 mM CsCl2, 5 mM EGTA and 5 mM HEPES and was adjusted to pH 7.4 with CsOH. Microelectrodes were made of borosilicate glass with a puller (P-87, Sutter Instrument Co, Novato, CA, USA) and were heat-polished using a microforge (MF-83, Narishige, Tokyo, Japan). The resistances of microelectrodes ranged from 1.0 to 2.0 MΩ when filled with a recording solution. Voltage clamp was generated by Axopatch 200B amplifier (Axon Instruments, Foster City, CA) and controlled using pClamp software 10.2. The series-resistance was compensated usually to ~80%. The *I*_Na_ was normalized by cell capacitance to obtain current density. The standard voltage-clamp protocols are presented with the data and have been previously described [19].

### Flow cytometry

HEK293 cells were transiently transfected with HA-tagged WT-Q1077del, HA-tagged R1512W/Q1077del, HA-tagged R1512W/Q1077del+MEX, HA-tagged R1512W/Q1077del/H558R, respectively. After 48 hours of transfection with or without mexiletine treatment, the cells were harvested by incubation with 0.5 mM EDTA-PBS for 10 min at 37°C and washed with RPMI 1640 supplemented with 1 mM EDTA (pH 7.4), 3% FCS, and 0.02% azide (staining medium). The FITC-conjugated anti-HA antibody (Sigma-Aldrich) incubations were performed in a staining medium at 4°C and then washed by PBS supplemented with 1 mM EDTA (pH 7.4) and 1% FCS. The stained cells were examined as previously described [20] for quantitative plasma membrane expression with FACSCalibur (BD Biosciences, San Jose, CA).

### Statistical analysis

Data are shown as the mean value with bars representing the standard error of the mean (S.E.M.). Determinations of statistical significance were performed using a Student *t* test for comparisons of two groups or using analysis of variance (ANOVA) for comparing multiple groups. P < 0.05 after Bonferroni correction was considered statistically significant.

## Results

### Current expression of R1512W in Q1077del and Q1077 background

**Figure 1.**
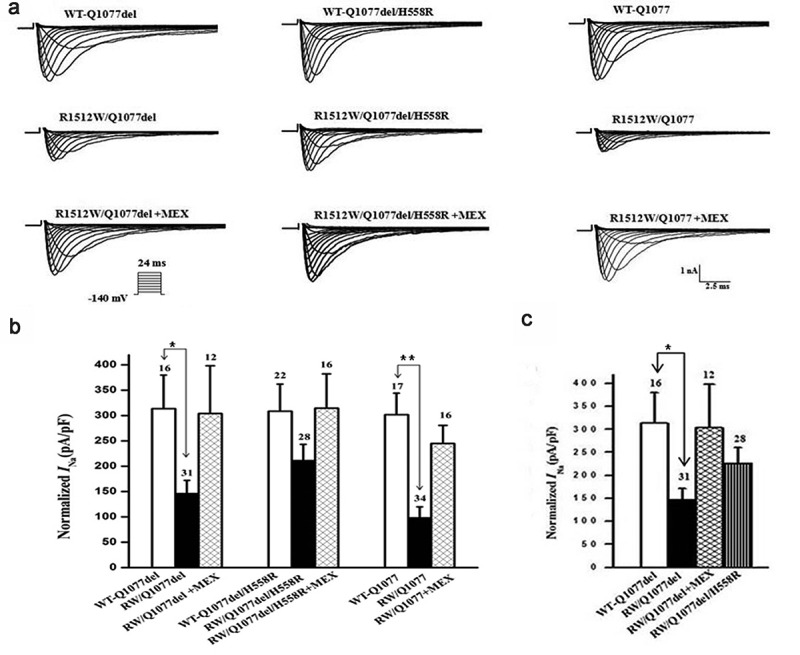
Expression defect for R1512W channels in Q1077del and Q1077 backgrounds and rescue for R1512W/Q1077del by H558R and mexiletine (MEX). (a) Whole-cell current traces from representative R1512W and WT channels in Q1077del and Q1077 backgrounds and R1512W/Q1077del with MEX or H558R. (b) Summary of Na^+^ current (*I*_Na_) density in R1512W and WT channels in Q1077del and Q1077 backgrounds and R1512W/Q1077del with MEX or H558R. (c)Comparison of Na^+^ current (*I*_Na_) density in R1512W/Q1077del, R1512W/Q1077del with MEX, R1512W/Q1077del/H558R and WT channels. The current amplitude was normalized to the membrane capacitance for each cell. *p value below 0.05/9 = 0.0055 (Bonferroni correction) indicates the *I*_Na_ density was significantly different compared R1512W without MEX to with MEX and WT in Q1077del background. **p < 0.0055 indicates the *I*_Na_ density was significantly different compared R1512W without MEX to with MEX and WT in Q1077 background. The *I*_Na_ density of R1512W was not significantly different compared to WT in Q1077del/H558R background

WT and R1512W mutant channels in the two common splice variant backgrounds Q1077del and Q1077 were voltage-clamped 24 hours after transient transfection with equal amounts of cDNA. Mean *I*_Na_ density for WT and mutant channels were compared for experiments performed on the same day in order to reduce variability. Representative examples of macroscopic *I*_Na_ traces for WT and mutant channels are shown in Figure 1a, with summary data given in Figure 1b and Table 1. In the Q1077del background, the mean current density of the R1512W mutant channel was −139 pA/pF, which was a significant decrease in comparison to WT channels (Figure 1b and Table 1, p < 0.005). In the Q1077 background, the R1512W mutant channel had a mean current density of −95 pA/pF, also showing a significant difference compared with the WT channel (Figure 1b and Table 1, p < 0.005). The partial expression defect for the R1512W mutant channel was profound in both splice variant background and this defect was even greater in the Q1077 background.

**Table 1.** Voltage-dependent gating parameters of each group in a heterologous expression system

	V_1/2_, mV		Recovery		
	Activation	Inactivation	τ_f_	τ_s_	A_s_, %
WT-Q1077del	46.8 ± 2.1 (18)	−83.3 ± 5.2 (11)	2.1 ± 0.6	47.2 ± 7.7	24.5 ± 1.4 (19)
WT-Q1077	45.4 ± 1.8 (16)	−82.9 ± 3.1 (10)	2.4 ± 0.4	52.2 ± 3.4	22.3 ± 1.5 (13)
RW/Q1077del	45.8 ± 1.6 (24)	−84.4 ± 8.9 (11)	2.6 ± 0.3	48.1 ± 5.0	21.8 ± 2.0 (16)
RW/Q1077	45.2 ± 1.4 (25)	−82.4 ± 8.1 (10)	3.5 ± 0.7^#^	82.9 ± 6.9^#^	24.4 ± 2.2 (15)
RW/Q1077del+MEX	46.4 ± 1.6 (12)	−83.0 ± 5.9 (13)	2.2 ± 0.4	48.4 ± 4.8	21.5 ± 1.6 (10)
RW/Q1077del/H558R	46.7 ± 1.5 (20)	−81.8 ± 2.58 (13)	2.2 ± 0.6	47.9 ± 3.6	22.0 ± 1.2 (17)

The fitted values of voltage-dependent gating parameters represent the mean SEM for number of experiments in the parentheses. These parameters were obtained from fitting the individual experiments to the appropriate model equations. For the Boltzmann fits the parameters of V_1/2_ are the midpoint of activation and inactivation. # p < 0.05 indicates the time constants of recovery were significantly different compared R1512W and WT in Q1077 background.

### Rescuing effects of mexiletine and H558R on R1512W mutant channels

HEK-293 cells expressing WT or R1512W mutant channels were incubated for 24 hours with 500 μM mexiletine at room temperature. Currents were measured after washout of mexiletine. Mean *I*_Na_ density for mutant channels both in Q1077del and Q1077 splice variant background were increased dramatically and significantly after incubation, compared with mutant channels without mexiletine incubation (Figure 1b and Table 1). Moreover, we expressed the most common SCN5A polymorphism H558R in the more predominant splice variant background Q1077del and measured whole-cell voltage-clamp for WT-Q1077del, R1512W/Q1077del, and R1512W/Q1077del/H558R in HEK293 cells. Interestingly, when the mutation was expressed in the presence of the H558R polymorphism, the sodium currents recorded were comparable to those recorded from WT sodium channels (Figure 1b and Table 1), suggesting that the polymorphism restored the current of this mutated channel. Therefore, both mexiletine and the H558R polymorphism restored the decreased sodium currents of mutant channels in the Q1077del splice variant background to nearly WT levels (Figure 1c).

### Voltage-dependent gating properties of R1512W in Q1077del and Q1077 background

**Figure 2.**
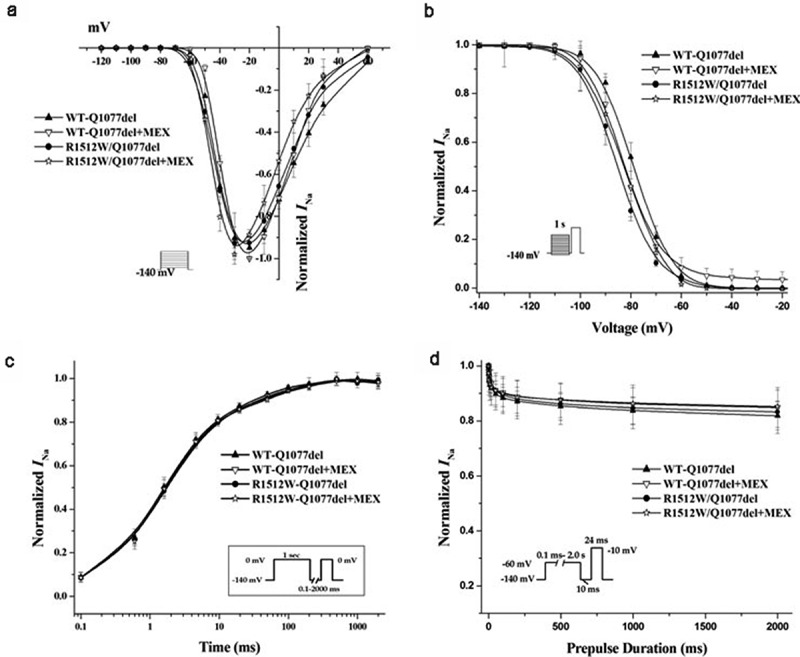
Voltage-dependent gating for R1512W and WT channels in Q1077del with and without MEX. (a) Voltagedependence of activation for R1512W and WT with and without MEX. The voltage clamp was 24 ms step depolarization to different potentials in increments of 10 mV from holding potential of – 140 mV (see insert). (b) Steady-state availability from inactivation for R1512W and WT with and without MEX. (c) Recovery from inactivation for R1512W and WT channels with time on a log scale to better show the early time course of recovery. (d) Intermediate inactivation for R1512W and WT channels with and without MEX. The activation and inactivation midpoints, intermediate inactivation and recovery from inactivation are not significantly difference between WT and mutant in Q1077del splice variant background. Insects: diagrams of voltage protocols. Values are means ± SE; n and fit parameters are given in Table 1

**Figure 3.**
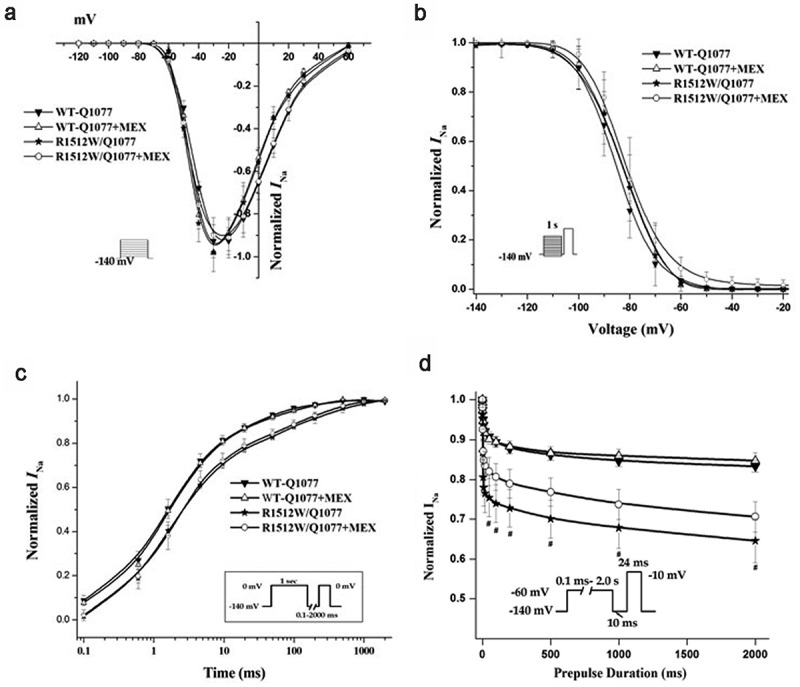
Voltage-dependence of activation (a), inactivation (b), recovery from inactivation (c), and intermediate inactivation (d) for R1512W and WT channels with and without MEX as in Figure 3 except in the Q1077 background. The activation and inactivation midpoint are not difference between WT and mutant channels. However, the slower time constants of recovery and enhanced intermediate inactivation were showed for R1512W compared with WT with and without MEX in the Q1077 splice variant background. # p < 0.05 indicates intermediate inactivation were significantly different compared R1512W and WT in Q1077 background

The voltage-dependent gating properties of the R1512W mutant channels in both splice variant background are compared with those of WT channels in Figures 2 and 3; summary data for the fitted kinetic parameters are shown in Table 1. The activation and inactivation of the channels were the same for both WT backgrounds and the mutation in both backgrounds (Figures 2 and 3). The recovery from inactivation was not significantly different between WT and mutant in Q1077del splice variant background. However, slower time constants of recovery were observed for R1512W than that for WT in the Q1077 background (Figure 3c). Finally, a protocol was designed to measure the component of current inactivated in a prepulse that cannot recover quickly. This inactivation, which is sometimes called intermediate, slow, and ultraslow inactivation, depending on the length of the prepulse, will be referred to collectively as slow inactivation. Slow inactivation was not significantly different between WT and mutant in the Q1077del splice variant background (Figure 2d). However, slow inactivation was significantly greater in mutant than in WT channels, in the Q1077 splice variant background (Figure 3d).

### Cell surface expression of Na channel protein in Q1077del background with and without drug treatment or H558R polymorphism

**Figure 4.**
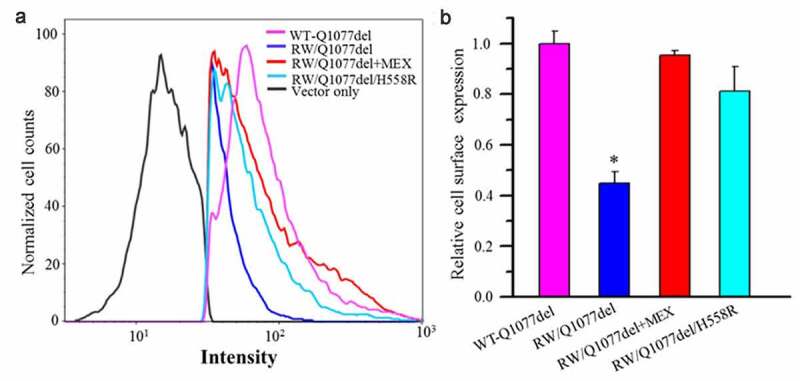
Flow cytometric analysis of cell surface expression of the HA-tagged SCN5A WT and mutant channels. HEK293 cells transiently transfected with HA-tagged WT-Q1077del, HA-tagged R1512W/Q1077del, HA-tagged R1512W/Q1077del+MEX, HA-tagged R1512W/Q1077del/H558R, respectively. After forty-eight hours of transfection with or without MEX treatment, the cells were harvested and stained by FITC-conjugated anti-HA antibody for quantitative the plasma membrane expression of HA-tagged channels by flow cytometry. (a) The Intensity of HA-tagged WT-Q1077del, HA-tagged R1512W/Q1077del, HA-tagged R1512W/Q1077del+MEX and HA-tagged R1512W/Q1077del/H558R. (b) Quantitation data to show the percentage of counted cells which the plasma membrane expressed HA-tagged WT-Q1077del, HA-tagged R1512W/Q1077del, HA-tagged R1512W/Q1077del+MEX, HA-tagged R1512W/Q1077del/H558R, respectively. *p < 0.001, compared with WT

We used flow cytometry to detect the channel cell surface expression of HA-tagged R1512W/Q1077del and WT-Q1077del. The R1512W mutation was noted to decrease cell surface expression of Na channels compared with WT (Figure 4). Furthermore, we used flow cytometry to discover whether drug treatment or H558R polymorphism could affect the cell surface expression. As expected, both previous incubation with mexiletine and H558R polymorphism could dramatically improve cell surface expression of the mutant channels (Figure 4b), consistent with the current expression data shown in Figure 1c.

## Discussion

In the present study, we found that the SCN5A mutation R1512W exhibited significantly decreased peak sodium currents. This loss-of-function effect is likely caused by a trafficking defect with incomplete expression relative to WT channels and the severity of the defect depends on the splice variant background in which it is expressed and tended to be worse in the Q1077 variant. The antiarrhythmic drug mexiletine improved the expression of the mutant channel and restored the current density of R1512W after incubation to WT levels. Interestingly, when the H558R polymorphism co-existed with the R1512W mutation on the same gene, the current density also was increased back to WT levels.

Previously, most of the SCN5A arrhythmia mutations causing loss-of-function were studied in only one of the splice variant backgrounds. Functional studies of WT sodium channels showed no significant difference between Q1077del and Q1077 background in functional properties [2]. However, when some mutations are introduced into these two splice backgrounds, electrophysiological discrepancies of Na^+^ channels were found. Wang et al. discovered one in-frame deletion allele (delAL586-587) and two missense variants (R680H, V1951L) that exhibited increased persistent sodium current only when expressed in the context of the common splice variant Q1077del [21]. We previously reported that SCN5A mutation S1787N significantly increased late *I*_Na_ only in the splice variant Q1077del [18]. We also discovered a partial expression defect for SCN5A mutation G1406R depends on splice variant background Q1077 [3]. So we hypothesized that the splice variant background might also be important for the SCN5A mutation R1512W with loss-of-function. This study shows that the partial expression defect for R1512W tended to be more severe in the Q1077 background. The voltage-dependent gating of mutated R1512W channels differed from WT in a way that would favor decreased peak current, but only when expressed in the Q1077 splice variant. This result again demonstrates that the splice variant background can be important in determining the functional properties of a mutation in heterologous expression systems. Considering that R1512W was observed in both BrS patients and healthy Hispanics, we noted the differences in penetrance or expressivity for SCN5A loss-of-function mutations [22]. The background sodium current density for sodium channel mutations may vary within the normal population. This could be one explanation for the clinical observations of the same single SCN5A missense mutation leading to different phenotypes, depending on the different genetic background.

For the “loss-of-function” found in the SCN5A mutation, mexiletine tended to restore the decreased peak *I*_Na_ by rescuing the cell surface localization of the protein [23]. In our study, SCN5A-R1512W showed approximately a 50% reduction in peak *I*_Na_ that was rescued by mexiletine. Valdivia et al. reported M1766L as the first SCN5A mutation expression defect rescued by mexiletine [24]. In our previous study, mexiletine rescued a mixed biophysical phenotype of the cardiac sodium channel arising from the SCN5A mutation N406K [20]. While mexiletine is known to correct Na_v_1.5 trafficking defects, the A124D and V1378M mutants did not react in the same way [25]. Mexiletine could partially restore the current density of V1378M but had no effect on the current density of A124D. As a Class Ib antiarrhythmic drug, mexiletine binds to Na_v_1.5 channels at the local anesthetic binding site in the sixth transmembrane segment of the fourth homologous domain [26]. Moreau et al. hypothesized that when mexiletine binds to V1378M channels in the endoplasmic reticulum, it acts as a molecular chaperone to allow the export of the retained channels [25]. On the other hand, the A124D mutation in the N-terminus may be too far away from the local anesthetic binding site for mexiletine to be effective. This suggests that the effectiveness of mexiletine to rescue trafficking defects may depend on the location of the mutation.

Several reports demonstrated that the H558R polymorphism mitigated the gain-of-function defects caused by several gain-of-function SCN5A mutations underlying LQT3 syndrome [14,15,27]. Moreover, H558R mitigated the decreased *I*_Na_ produced by loss-of-function mutations. Concerning BrS, H558R increases the peak *I*_Na_ density mainly by restoring their defective trafficking [28–32]. Furthermore, restoration was apparent when the mutation and the polymorphism were present either in the same or in a different SCN5A allele [9,30,32]. The trafficking restoration produced when the polymorphism and the mutation are present in the same allele has empirically been attributed to a stabilizing effect produced by H558R such that the polymorphism restores the proper protein folding overcoming the quality controls before protein trafficking [33].

The biophysical properties of R1512W were previously characterized by Zheng et al. [11] found in a Chinese SUNDS victim. They reported decreased peak *I*_Na_ in SCN5A-Q10771del background at pH7.4 and an even greater reduction in peak *I*_Na_ at pH 7.0 compared to WT. Our study and Cheng’s study provided evidence that the splice variant background, the presence of H558R polymorphism, as well as environmental factors such as acidosis could affect the molecular phenotype of SCN5A.

There are some limitations to the present study. Firstly, the electrophysiological data were generated by in vitro experiments using HEK293 cells expressing the SCN5A, which is somewhat different from the physiological environment in human cardiomyocytes. The SCN5A complex in native tissue has many more components that we did not coexpress in the heterologous cell model, and some interacting proteins found in the native heart could have modulatory roles. Our experiments in the heterologous system only suggest a possible biophysical phenotype that may lead to the clinical syndromes, but further studies in a more native environment of cardiomyocytes are necessary to describe the full pathogenetic pathway. Secondly, it should be noted that the doses of mexiletine used in this study for rescue in the heterologous system are greater than clinically achievable levels. Further study would be required to better assess the clinical implications of these findings.

## Conclusions

In conclusion, this study characterized the electrophysiological function of the SCN5A missense mutation R1512W and showed partial expression defect in the two common splice variant backgrounds, Q1077del and Q1077, and the defect being worse in the Q1077 splice variant. The most common polymorphism H558R and the anti-arrhythmic drug, mexiletine can increase the expression level of this BrS mutation to WT level. These findings provide further evidence that the splice variant background affects the molecular phenotype with implications for the clinical phenotype. Both expression defect and kinetic abnormalities may contribute to a “loss-of-function” phenotype in the same mutation.
